# Associations of Body Composition Measurements with Serum Lipid, Glucose and Insulin Profile: A Chinese Twin Study

**DOI:** 10.1371/journal.pone.0140595

**Published:** 2015-11-10

**Authors:** Chunxiao Liao, Wenjing Gao, Weihua Cao, Jun Lv, Canqing Yu, Shengfeng Wang, Bin Zhou, Zengchang Pang, Liming Cong, Hua Wang, Xianping Wu, Liming Li

**Affiliations:** 1 Department of Epidemiology and Biostatistics, School of Public Health, Peking University, Beijing, China; 2 Qingdao Center for Diseases Control and Prevention, Qingdao, China; 3 Zhejiang Center for Disease Control and Prevention, Hangzhou, China; 4 Jiangsu Center for Disease Control and Prevention, Nanjing, China; 5 Sichuan Center for Disease Control and Prevention, Chengdu, China; McMaster University, CANADA

## Abstract

**Objectives:**

To quantitate and compare the associations of various body composition measurements with serum metabolites and to what degree genetic or environmental factors affect obesity-metabolite relation.

**Methods:**

Body mass index (BMI), waist circumference (WC), lean body mass (LBM), percent body fat (PBF), fasting serum high density lipoprotein cholesterol (HDL-C), low density lipoprotein cholesterol (LDL-C), triglycerides (TG), total cholesterol (TC), glucose, insulin and lifestyle factors were assessed in 903 twins from Chinese National Twin Registry (CNTR). Homeostasis model assessment of insulin resistance (HOMA-IR) was calculated from fasting serum glucose and insulin. Linear regression models and bivariate structural equation models were used to examine the relation of various body composition measurements with serum metabolite levels and genetic/environmental influences on these associations, respectively.

**Results:**

At individual level, adiposity measurements (BMI, WC and PBF) showed significant associations with serum metabolite concentrations in both sexes and the associations still existed in male twins when using within-MZ twin pair comparison analyses. Associations of BMI with TG, insulin and HOMA-IR were significantly stronger in male twins compared to female twins (BMI-by-sex interaction p = 0.043, 0.020 and 0.019, respectively). Comparison of various adiposity measurements with levels of serum metabolites revealed that WC explained the largest fraction of variance in serum LDL-C, TG, TC and glucose concentrations while BMI performed best in explaining variance in serum HDL-C, insulin and HOMA-IR levels. Of these phenotypic correlations, 64–81% were attributed to genetic factors, whereas 19–36% were attributed to unique environmental factors.

**Conclusions:**

We observed different associations between adiposity and serum metabolite profile and demonstrated that WC and BMI explained the largest fraction of variance in serum lipid profile and insulin resistance, respectively. To a large degree, shared genetic factors contributed to these associations with the remaining explained by twin-specific environmental factors.

## Introduction

The obesity epidemic has been a worldwide phenomenon, with 62% of the world's obese individuals living in developing countries [1]. Obesity represents a major public health challenge as it promotes dyslipidemia [2], hyperglycemia and insulin resistance [3] and is associated with a significant rise in comorbidities risk, including metabolic syndrome (MS), cardiovascular disease(CVD) and type 2 diabetes mellitus (T2DM), leading to increased disease burden and higher all-cause mortality [4].

Body mass index (BMI) is the most widely used method for the diagnosis of obesity and is correlated directly with the risk of comorbidities and mortality [5]. In addition, evidence from epidemiological studies has demonstrated the importance of abdominal obesity, assessed by waist circumference (WC), in predicting insulin resistance, dyslipidemia and other obesity-related health risk [6,7] and recent findings have indicated that WC is a stronger marker of health risk than is BMI [8]. Although BMI and WC are simple convenient measures for epidemiological studies, their validity in measuring adiposity has been questioned because they do not directly measure the amount of adipose tissue and could not differentiate between fat mass (FM) and lean body mass (LBM) [9]. Some studies have found that percent body fat (PBF), a more direct assessment of adiposity, to be a better discriminator of cardiovascular and abnormal serum metabolism than simple anthropometric parameters [10, 11], whereas others have found them to be equivalent [12, 13]. In the context of the efforts to control the contemporary epidemic of obesity and associated diseases, a full understanding of the relation between different measures of obesity and health risk is greatly needed.

It is also worth noting that body composition measurements and obesity-related metabolic phenotypes are both influenced by genetic factors. Previous twin studies showed moderate to high heritability of different body composition measurements including BMI, WC, and PBF [14–16] while heritability for serum lipids ranged from 48% to 62% [17]. As most previous studies [18–20] were unable to control for the individual genetic variability it was unknown whether associations between these measurements and serum metabolite levels were attributable to shared genetic vulnerabilities influencing both phenotypes. Twin design is seen as a useful method of controlling confounders in observational epidemiologic studies. Especially monozygotic (MZ) twins who are completely matched for any variations in the genetic background provide an extremely powerful control for genetic confounding factors. Using structural equation modeling methods, twin studies can further evaluate how genetic and/or environmental factors contribute to the relation between body composition measurements and serum metabolites.

Previous twin studies examining the relationship between various body composition measurements and serum metabolite levels were mainly conducted in western populations [21, 22]. There have been reports in Chinese adolescents and female adults but limited in one rural region [23, 24]. It is not clear whether the patterns of this previously reported association can be applied to adults in other parts of China.

Therefore, we aimed to quantitate the associations of three adiposity measurements (BMI, WC and PBF) and LBM with obesity-related health risks centered on fasting serum lipid, glucose, and insulin levels separately using a twin sample in 9 cities of China. Insulin resistance was defined according to homeostasis model assessment of insulin resistance (HOMA-IR)). Further, we extended current study by estimating genetic and environmental contributions to the associations of serum metabolites with adiposity measurements which have not previously been examined in Chinese adult people and that is not possible in a general population design.

## Methods

### Study sample

The participants belong to the Chinese National Twin Registry (CNTR), the first and largest population-based twin registry in China described in detail elsewhere [25]. Since its establishment in 2001, it has recruited 36,565 twin pairs (as of June 2014) from 9 provinces or cities in China, including Jiangsu, Zhejiang, Sichuan, Heilongjiang, Qinghai and Shandong province and Tianjin, Beijing and Shanghai city.

The analyses in this paper were based on a follow-up survey held from April to December 2013 among 1147 participants. The subjects were adult twins from four provinces covering 9 cities in Shandong, Zhejiang, Jiangsu and Sichuan province who completed an in-person questionnaire interview, a physical examination and a fasting blood biochemical test.

Pregnant female twins were excluded from participation. Twins were excluded from analyses if: (1) with a definitive diagnosis of medical diseases such as cancer, diabetes, cardiovascular heart disease, stroke and kidney disease; (2) treated with weight-, lipid- or glucose-lowering pharmacological agents. At last, a total of 903 individuals (385 completed twin pairs and 133 individuals) were eligible for this study.

Determination of zygosity was based on the information from questionnaires during the baseline investigation. Twins of different genders were directly classified as DZ. For twins of the same gender, a model was built according to age, gender and ‘whether they were as alike as two peas in a pod’. The model has been validated using DNA genotyping and found to be >90% accurate [26]. All participants provided their written informed consent and Biomedical Ethics Committee at Peking University, Beijing, China approved the study protocol.

### Body Composition measurements

Body composition measurements were expressed as BMI (kg/m^2^), WC (cm), PBF and LBM (kg). BMI was calculated as weight (kg)/height^2^ (m). Height was measured to the nearest 0.1 cm on a portable stadiometer while weight was measured to the nearest 0.1 kilograms using a digital balance (Body Composition Analyzer/Scale, TANITA, Tokyo, Japan). WC was measured three times at the level of the umbilicus to the nearest centimeter and the mean value was used in the analyses. PBF was determined by bioelectrical impedance (Body Composition Analyzer/Scale, TANITA). LBM was calculated by subtracting total body fat from total body weight. All investigators were trained and qualified for measurements.

### Biochemical measurements

Each sample was collected, processed, stored and transported in the same way across 9 cities. Venous blood samples were drawn from the study subjects after a 12-h fast. Blood samples were kept in the portable blood refrigerator of 4°C and subsequently centrifuged for 20 min in a tabletop refrigerated centrifuge at 2500 rpm. Identical processing procedures were rigorously controlled for at each testing period. Serum samples were frozen and stored at -20°C in the local health-center and were transported via cold chain system to central laboratory in Beijing and stored at -80°C within one month.

Serum total cholesterol (TC) and triglycerides (TG) were measured by the enzymatic colorimetric method (Roche, Basel, Switzerland). Direct methods were applied to assess high density lipoprotein cholesterol (HDL-C) and low density lipoprotein cholesterol (LDL-C) (Roche, Basel, Switzerland). A modified hexokinase enzymatic method was used to detect glucose (Glu) (Roche, Basel, Switzerland), and serum insulin was measured by chemiluminescence immunoassay (CLIA) on the ADVIA Centaur immunoassay system. Insulin resistance was estimated according to homeostasis model assessment (HOMA-IR): HOMA-IR = [fasting glucose (mmol/l) × insulin (U/ml)]/22.5 [27]. To minimize the effects of assay variability, samples from each twin pair were analyzed using the same assay.

### Assessment of covariates

We obtained covariates from questionnaire, including sociodemographic characteristics (age, sex, region, and social economic status) and lifestyle behaviors (tobacco smoking, alcohol drinking and physical activity). Region was assessed by place of living, divided into four categories (Shandong, Zhejiang, Jiangsu, Sichuan province). Social economic status (low, medium, high) was derived from five questions including occupation, level of education, per capita monthly expenditure, per capita monthly food expenditure and per capita housing area. Tobacco smoking was coded into three categories (never, former, current) according to participants’ responses to ‘Do you smoke’. Alcohol drinking status was similarly defined depending on their responses to ‘Do you drink alcohol’. Participants’ exercise activities on occupation, transportation, daily life and leisure time were assigned a metabolic equivalent task (MET) value, using the Compendium of Physical Activities by Ainsworth et al. [28], after which the MET value was classified into three levels (low, medium and high) according to the International Physical Activity Questionnaire (IPAQ) group to represent the levels of physical activity.

### Statistical methods

We compared epidemiological, physical and biochemical characteristics between male and female twins. P values were corrected for the correlation between co-twins using multinomial logistic regression for categorical variables and mixed-effects models for continuous variables. Pearson’s correlation coefficients were used to examine the relation between anthropometric measures. Regression models and structural equation models were used to examine the associations of different body composition measurements with serum metabolite profile.

### Linear regression analysis

First, in the entire sample treating twins as separate individuals, sex-specific mixed-effect linear regression models with a random intercept for each twin pair to account for twin clustering [29] were performed to examine the relationship between multiple body composition measurements (explanatory variables) and serum lipid, glucose, insulin and HOMA-IR levels (outcome variables), adjusting for age(continuous), zygosity(MZ or DZ), region (Shandong, Zhejiang, Jiangsu, or Sichuan province), social economic status (low, medium, or high), smoking status (never, former, or current smoker), drinking status (never, former, or current drinker), and physical activity (low, medium, or high). Secondly, to investigate whether these associations were confounded by genetic factors, we applied co-twin regression analyses within MZ twin pairs. The within-pair approach automatically takes into account shared familial and environmental influences. These within-pair analyses were further stratified by sex to estimate the relation between measures of body composition and levels of serum metabolites separately for male and female MZ twins. Next, we tested the interaction between sex and each of the body composition measurements on the serum metabolite measures. Significance of these interactions demonstrates that the associations between measures of body composition and levels of serum metabolites differ as a function of gender. Further analyses focused on BMI, WC and PBF as measures of adiposity. In order to make a comparison between effects of different adiposity measurements on serum metabolite levels, we standardized all the adiposity measurements into z-scores for each linear regression model with R^2^ values calculated. A z-score was calculated for each measurement as the observed value minus the mean value, divided by the standard deviation within each stratum of age- and gender-group [24]. It represents the change in a variable by units of its standard deviation.

All the serum metabolites were handled after logarithmic transformation in the regression analyses. Robust standard error and confidence intervals for estimates have been produced. All the statistical analyses were performed with Stata statistical software (release 12.0; Stata Corporation, College Station, TX). P-values are two-sided, and statistical significance was assumed at *P*<0.05.

### Bivariate structural equation modeling

The phenotypic correlation between adiposity measurements and serum metabolites, as well as the extent to which it is attributable to additive genetic, common environmental, or unique environmental variance may be estimated using bivariate structural equation models. Using the approach, the relative contributions of additive genetic, common environmental, and unique environmental components on the observed associations between adiposity measurements and serum metabolites may be quantified, respectively.

We fitted a saturated univariate additive genetic/common environment/unique environment model (ACE model) for adiposity measurements and serum metabolites mentioned above. Nested model for which C was equated to zero was also fitted and Akaike Information Criterion (AIC) was used for comparison of goodness of fit of the models. Next, based on the best fit model we fitted the bivariate Cholesky decomposition models to calculate genetic (r_G_) and unique environmental correlations (r_E_) between adiposity measurements and serum metabolites and 95% CIs. All model fitting analyses and maximum-likelihood parameter estimates were performed in OpenMx (Version 1.4) with logarithmic transformed serum metabolites and raw data of adiposity measurements and all variance components were estimated with inclusion of age, sex, region, social economic status, smoking status, drinking status and physical activity as covariates in the models.

## Results

### Sample characteristics

A total of 903 individuals including 235 complete MZ twin pairs and 150 complete DZ twin pairs were eligible for our analyses. The epidemiological characteristics of the participants are summarized in Table 1. This study population consisted of 583 male twins and 320 female twins with a mean age of 46.1 and 44.0, respectively. One-half of men were current smokers and the prevalence of alcohol drinking among men was slightly lower at 47.2% whereas these figures were negligible in women. However, levels of physical activity and SES did not differ by sex. Their body composition and biochemical characteristics are presented in Table 2. Men and women had comparable levels of serum HDL-C, TC, insulin and HOMA-IR; besides, men were expectedly heavier (24.9 vs. 23.6 kg/m^2^) and had a larger waist circumference (87.9 vs. 81.5cm) while lower percent body fat (23.4%vs. 32.2%) than women(all *P*<0.001).

**Table 1:** Epidemiological characteristics of the 903 Chinese adult twins.

	All twins(n = 903)	Male twins(n = 583)	Female twins(n = 320)	*P* value ^a^
Age(years), mean(SD)	45.4(13.1)	46.1(12.6)	44.0(13.7)	0.779
Zgosity(MZ), n (%)	548(60.7)	358(61.4)	190(59.4)	0.919
Region, n (%)				
Qingdao	175(19.4)	132(22.6)	43(13.4)	-
Jiangsu	358(39.7)	213(36.5)	145(45.3)	0.005
Sichuan	99(11.0)	68(11.7)	31(9.7)	0.338
Zhejiang	271(30.0)	170(29.2)	101(31.6)	0.028
SES, n (%)				
Low	190(21.0)	123(21.1)	67(20.9)	-
Medium	632(70.0)	402(69.0)	230(71.9)	0.797
High	81(9.0)	58(9.9)	23(7.2)	0.298
Smoking status, n (%)				
Never	519(57.5)	201(34.5)	318(99.4)	-
Current	293(32.5)	292(50.1)	1(0.3)	<0.001
Former	91(10.1)	90(15.4)	1(0.3)	<0.001
Drinking status, n (%)				
Never	579(64.1)	294(50.4)	285(89.1)	-
Current	309(34.2)	275(47.2)	34(10.6)	<0.001
Former	15(1.7)	14(2.4)	1(0.3)	0.013
Physical activity level, n (%)				
Low	56(6.2)	42(7.2)	14(4.4)	-
Medium	215(23.8)	135(23.2)	80(25.0)	0.108
High	632(70.0)	406(69.6)	226(70.6)	0.123

SD, standard deviation; MZ, monozygotic; SES, social economic status.

n = 903 individuals (385 twin pairs and 133 individuals).

^a^ P values were corrected for the correlation between co-twins using multinomial logistic regression for categorical variables and mixed-effects models for continuous variables.

**Table 2:** Body composition and biochemical characteristics of the 903 Chinese adult twins.

	All twins		Male twins		Female twins		
	N	Mean ^a^	N	Mean	N	Mean	*P* value ^b^
BMI((kg/m^2^)	903	24.4(24.2,24.7)	583	24.9(24.6,25.2)	320	23.6(23.2,24.1)	<0.001
WC(cm)	903	85.6(84.9,86.3)	583	87.9(87.0,88.7)	320	81.5(80.4,82.6)	<0.001
PBF	879	26.5(26.0,27.0)	564	23.4(22.8,23.9)	315	32.2(31.4,32.9)	<0.001
LBM(kg)	879	46.7(46.1,47.3)	564	51.6(51.0,52.2)	315	37.8(37.4,38.3)	<0.001
HDL-C(mmol/L)	903	1.4(1.2,1.6)	583	1.4(1.2,1.6)	320	1.5(1.3,1.7)	<0.001
LDL-C(mmol/L)	903	2.1(1.8,2.6)	583	2.2(1.8,2.6)	320	2.1(1.7,2.5)	0.542
TG(mmol/L)	903	1.3(0.9,1.9)	583	1.3(0.9,2.0)	320	1.3(0.9,1.3)	0.027
TC(mmol/L)	903	4.7(4.1,5.4)	583	4.7(4.1,5.4)	320	4.7(4.1,5.3)	0.731
Glucose (mmol/L)	903	5.2(4.9,5.7)	583	5.3(4.9,5.9)	320	5.1(4.8,5.5)	0.027
Insulin(pmol/L)	856	56.4(37.9,87.3)	559	54.3(35.2,86.6)	297	60.7(41.8,92.8)	0.673
HOMA-IR	856	2.0(1.2,3.1)	559	1.9(1.2,3.2)	297	2.0(1.4,3.0)	0.744

BMI, body mass index; WC, waist circumference; PBF, percentage body fat; LBM, lean body mass; HDL-C, high density lipoprotein cholesterol; LDL-C, low density lipoprotein cholesterol; TG, triglycerides; TC, total cholesterol; HOMA-IR, homeostasis model assessment of insulin resistance.

n = 903 individuals (385 twin pairs and 133 individuals) and sample size vary due to missing values.

^a^ Data are reported as mean (95%CI) for body composition measurements and median (interquartile range) for biochemical measures.

^b^ *P* values were corrected for the correlation between co-twins using mixed-effects models for continuous variables.

### Associations of body composition measurements with serum lipid, glucose and insulin profile

The Pearson’s phenotypic correlations between different body composition measurements are presented in S1 Table. Correlations estimated highest at 0.825 (95%CI 0.763–0.867) between BMI and WC while lowest at -0.266(95%CI -0.319– -0.211) between PBF and LBM. The associations between various body composition measurements and serum lipid, glucose, insulin and HOMA-IR levels separated by gender are showed in Table 3. In both genders, a negative relationship was observed between adiposity measurements and HDL-C level whereas the remaining serum metabolites, including LDL-C, TG, TC, glucose, insulin and HOMA-IR levels were positively associated with the three adiposity measurements. In stark contrast, LBM only showed a significant association with serum insulin and HOMA-IR concentrations in female twins whereas additionally exerted an influence on HDL-C and TG in male twins.

**Table 3:** Random-intercept regression analyses of body composition measurements and serum metabolites in 903 Chinese adult twins stratified by gender, treating twins as individuals.

	HDL-C(mmol/L)	LDL-C(mmol/L)	TG(mmol/L)	TC(mmol/L)	Glucose (mmol/L)	Insulin(pmol/L)	HOMA-IR
	*β*(95%*CI*)	*β*(95%*CI*)	*β*(95%*CI*)	*β*(95%*CI*)	*β*(95%*CI*)	*β*(95%*CI*)	*β*(95%*CI*)
Male							
BMI(kg/m^2^)	-0.007(-0.009,-0.005)***	0.008(0.005,0.010) ***	0.032(0.027,0.037) ***	0.004(0.002,0.006) ***	0.004(0.003,0.006) ***	0.044(0.038,0.050) ***	0.048(0.042,0.054) ***
WC(cm)	-0.002(-0.003,-0.001) ***	0.003(0.002,0.004) ***	0.010(0.007,0.013) ***	0.002(.0009,0.002) ***	0.002(.0009,0.002) ***	0.014(0.011,0.017) ***	0.015(0.012,0.019) ***
PBF	-0.003(-0.005,-0.002) ***	0.004(0.003,0.006) ***	0.018(0.014,0.021) ***	0.003(0.002,0.004) ***	0.003(0.002,0.004) ***	0.022(0.019,0.026) ***	0.025(0.021,0.029) ***
LBM(kg)	-0.004(-0.005,-0.003) ***	0.001(-.0006,0.003)	0.012(0.008,0.015) ***	.0007(-.0005,0.002)	.0009(-.0002,0.002)	0.015(0.011,0.020) ***	0.015(0.011,0.020) ***
Female							
BMI(kg/m^2^)	-0.004(-0.006,-0.001) **	0.007(0.003,0.011) ***	0.017(0.011,0.024) ***	0.004(0.001,0.006) **	0.003(0.001,0.005) **	0.034(0.028,0.041) ***	0.038(0.031,0.045) ***
WC(cm)	-0.001(-0.002,-.0000)*	0.003(0.001,0.004) ***	0.006(0.003,0.009) ***	0.002(.0005,0.002) **	0.001(.0004,0.002) **	0.012(0.010,0.015) ***	0.013(0.011,0.016) ***
PBF	-0.001(-0.003,0.002)	0.004(0.002,0.006) ***	0.010(0.006,0.014) ***	0.003(0.001,0.004) ***	0.002(.0006,0.002) **	0.019(0.016,0.023) ***	0.021(0.017,0.025) ***
LBM(kg)	-0.003(-0.006,.0004)	0.002(-0.002,0.006)	0.003(-0.003,0.010)	-.0000(-0.003,0.003)	0.002(-.0001,0.003)	0.013(0.006,0.021) **	0.015(0.007,0.024) ***

Abbreviations are the same as in Table 2.

All regression models were adjusted for age, zygosity, region, social economic status, smoking status, drinking status and physical activity.

*p<0.05

**p<0.01

***p<0.001

In analyses controlling for genetic effects within 235 MZ twin pairs, associations of various body composition measurements with serum metabolite levels are presented in Table 4. In the whole MZ twins, most adiposity measurements had a significant association with serum metabolite levels independent of genetic influence, and similar pattern was seen between LBM and serum HDL-C, TG, glucose, insulin and HOMA-IR. Tests for statistically difference in association of different body composition measurements with serum metabolite levels between male and female MZ twins supported a sex-specific role of BMI on TG, insulin and HOMA-IR (BMI-by-sex interaction p = 0.043, 0.020 and 0.019, respectively). Stratified analyses by sex were slightly hampered by reduced statistical power. Nevertheless, the same pattern of these associations were obvious within MZ male twins but not within MZ female twins. These relationship were steeper in male twins than in female twins (S2 Table). Further analyses focused on WC, PBF, and BMI as measures of adiposity. As shown in Table 5, in general, the proportion of the variance in serum metabolite levels explained by adiposity varied by each metabolite and adiposity measurement (3–28%) and adiposity measurements in our models with larger β coefficients had relatively higher R^2^ values accounting for more variability of the serum metabolites. Among these adiposity measurements, BMI explained highest for variance in HDL-C, insulin and HOMA levels while WC explained highest for variance in levels of LDL-C, TG, TC and glucose. When 2 factor measures were examined (WC and BMI; WC and PBF), the R^2^ estimates did not change appreciably from those for single factor measures.

**Table 4:** Fixed-effect regression analyses of body composition measurements and serum metabolites within 235 MZ twin pairs; genders combined.

	HDL-C(mmol/L)		LDL-C(mmol/L)		TG(mmol/L)		TC(mmol/L)		Glucose (mmol/L)		Insulin(pmol/L)		HOMA-IR	
	*β*(95%*CI*)	P-value ^a^	*β*(95%*CI*)	P-value	*β*(95%*CI*)	P-value	*β*(95%*CI*)	P-value	*β*(95%*CI*)	P-value	*β*(95%*CI*)	P-value	*β*(95%*CI*)	P-value
BMI(kg/m^2^)	-0.008		0.007		0.029		0.004		0.004		0.045		0.05	
	(-0.012,-0.003)***	0.381	(0.002,0.012) **	0.775	(0.018,0.040) ***	0.043	(.0009,0.008) *	0.861	(0.001,0.008) **	0.755	(0.034,0.056) ***	0.020	(0.039,0.061) ***	0.019
WC(cm)	-0.002		0.003		0.009		0.002		0.002		0.013		0.015	
	(-0.003,-.0002)*	0.297	(0.001,0.004) ***	0.922	(0.006,0.013) ***	0.253	(.0007,0.003) **	0.598	(.0007,0.002) **	0.974	(0.009,0.017) ***	0.376	(0.010,0.019) ***	0.401
PBF	-0.002		0.004		0.012		0.003		0.002		0.02		0.022	
	(-0.004,.0006)	0.206	(0.002,0.006) **	0.938	(0.006,0.019) ***	0.072	(0.001,0.004) **	0.960	(.0005,0.003) *	0.517	(0.014,0.027) ***	0.183	(0.016,0.029) ***	0.161
LBM(kg)	-0.007		0.0008		0.017		< .001		0.002		0.025		0.028	
	(-0.010,-0.004) ***	0.147	(-0.003,0.005)	0.650	(0.009,0.025) ***	0.456	(-0.003,0.003)	0.704	(.0003,0.005) *	0.249	(0.017,0.033) ***	0.339	(0.018,0.037) ***	0.266

Abbreviations are the same as in Table 2.

All regression models were adjusted for age, sex, region, social economic status, smoking status, drinking status and physical activity.

^a^ P values for interaction between sex and each of the body composition measurements on the serum metabolite levels.

*p<0.05

**p<0.01

***p<0.001

**Table 5:** Fixed-effect regression analyses of the independent effect of adiposity measurements and serum metabolites within 235 MZ twin pairs; genders combined.

	HDL-C(mmol/L)		LDL-C(mmol/L)		TG(mmol/L)		TC(mmol/L)		Glucose (mmol/L)		Insulin(pmol/L)		HOMA-IR	
Adiposity measurements (z scores)	*β*(95%*CI*)	R^2^	*β*(95%*CI*)	R^2^	*β*(95%*CI*)	R^2^	*β*(95%*CI*)	R^2^	*β*(95%*CI*)	R^2^	*β*(95%*CI*)	R^2^	*β*(95%*CI*)	R^2^
BMI	-0.026		0.025		0.097		0.014		0.016		0.155		0.172	
	(-0.040,-0.011)***	0.086	(0.008,0.042) **	0.055	(0.061,0.133) ***	0.116	(0.002,0.026) *	0.028	(0.005,0.027) **	0.070	(0.118,0.192) ***	0.280	(0.133,0.211) ***	0.282
WC	-0.018		0.024		0.091		0.015		0.015		0.125		0.141	
	(-0.032,-0.005) **	0.063	(0.009,0.039) **	0.062	(0.059,0.124) ***	0.126	(0.005,0.026) **	0.040	(0.007,0.023) **	0.077	(0.089,0.162) ***	0.233	(0.102,0.179) ***	0.244
PBF	-0.014		0.025		0.081		0.016		0.011		0.131		0.143	
	(-0.031,0.003)	0.056	(0.010,0.040) **	0.057	(0.039,0.123) ***	0.093	(0.006,0.026) **	0.038	(0.003,0.019) *	0.052	(0.091,0.171) ***	0.224	(0.101,0.185) ***	0.224
WC+BMI														
WC	0.001		0.018		0.066		0.015		0.011		0.05		0.061	
	(-0.022,0.024)		(-0.007,0.044)		(0.022,0.110) *		(-0.005,0.036)		(-0.005,0.022)		(-0.014,0.113)		(-0.003,0.125)	
BMI	-0.026		0.009		0.038		0.0004		0.006		0.111		0.117	
	(-0.052,-0.002) *	0.086	(-0.020,0.038)	0.065	(-0.009,0.085)	0.137	(-0.022,0.023)	0.042	(-0.009,0.022)	0.078	(0.042,0.180) ***	0.291	(0.048,0.186) ***	0.296
WC+PBF														
WC	-0.018		0.014		0.074		0.009		0.014		0.046		0.091	
	(-0.036,.0003)		(-0.005,0.034)		(0.035,0.114) ***		(-0.006,0.024)		(0.003,0.024) *		(0.029,0.125) **		(0.041,0.141) ***	
PBF	-0.001		0.015		0.029		0.01		0.016		0.078		0.08	
	(-0.025,0.022)	0.075	(-0.003,0.033)	0.068	(-0.019,0.077)	0.136	(-0.005,0.024)	0.046	(-0.008,0.011)	0.071	(0.029,0.128) **	0.267	(0.029,0.131) **	0.272

Abbreviations are the same as in Table 2.

The z-score for a given adiposity measure was age- and sex-specific value.

All regression models were adjusted for age, sex, region, social economic status, smoking status, drinking status and physical activity.

*p<0.05

**p<0.01

***p<0.001

### Genetic and environmental contributions to the adiposity-serum metabolite associations

We further examined the relative contribution of genetic and environmental influences on the associations between adiposity measurements and serum metabolite levels. The following analyses included only paired same-sex twins.

Adiposity measurements and serum metabolites were all traits influenced by genetic factors. The additive genetic / unique environment (AE) model offered the best fit for all traits: dropping common environment (C) did not decrease the fit significantly with the P value for differences in model fits all bigger than 0.05. After adjustment for age, sex, region, smoking status, drinking status, physical metabolic equivalent value level and social economic status, the estimate of heritability for serum metabolites were moderate to high which ranged from 46.3% to66.3%. For adiposity measurements, heritability estimated lowest for WC at 59.7% while highest for PBF at 66.9% (S3 Table).

Bivariate analyses were focused only on the phenotypic associations of BMI with serum HDL-C, insulin and HOMA-IR and WC/PBF with serum lipid and glucose profile to reduce the number of combinations. Table 6 shows the genetic and environmental contributions to phenotypic correlations in this twin sample. The bivariate genetic analyses revealed high genetic correlation between PBF and TG (r_G_ = 0.80, 95%CI 0.43–0.99), modest genetic correlation between BMI and insulin (r_G_ = 0.69, 95%CI 0.47–0.99),BMI and HOMA-IR (r_G_ = 0.68, 95%CI 0.46–0.99), WC and TG (r_G_ = 0.55, 95%CI 0.19–0.99) as well as WC and glucose (r_G_ = 0.53, 95%CI 0.16–0.99), and a weak but significant association of PBF with TC (r_G_ = 0.44, 95%CI 0.03–0.80), and WC with LDL-C (r_G_ = 0.30, 95%CI 0.06–0.60). Significant contributions from unique environment effects were also found between all these phenotype pairs. Sixty-four to 81% of the total phenotypic correlations between each phenotype pair were determined by genetic factors, whereas the remaining 19–36% were due to unique environmental factors in this adult twin sample.

**Table 6:** Bivariate genetic analyses of the estimated genetic and environmental correlation coefficients for phenotype pairs.

Phenotype	r_G_ (95%CI)	r_E_ (95%CI)	c_G_	c_E_	%_G_	%_E_
BMI-Insulin	0.685 (0.472,0.999)	0.510 (0.407,0.600)	0.39	0.21	65.00	35.00
BMI-HOMA-IR	0.682 (0.461,0.999)	0.506 (0.403,0.596)	0.38	0.21	64.41	35.59
BMI-HDL-C	-0.323 (-0.999,0.999)	-0.246 (-0.360,-0.121)	0.20	0.09	68.97	31.03
WC-TG	0.552 (0.187,0.999)	0.346 (0.230,0.452)	0.31	0.15	67.39	32.61
WC-Glucose	0.530 (0.163,0.999)	0.225 (0.100,0.341)	0.31	0.09	77.50	22.50
WC-TC	0.320 (-0.029,0.662)	0.204 (0.080,0.322)	0.20	0.08	71.43	28.57
WC-LDL-C	0.303 (0.061,0.600)	0.229 (0.105,0.344)	0.19	0.08	70.37	29.63
PBF-TG	0.795(0.427,0.999)	0.286(0.163,0.400)	0.47	0.11	81.03	18.97
PBF-TC	0.442(0.032,0.801)	0.196(0.068,0.317)	0.29	0.07	80.56	19.44
PBF-LDL-C	0.414(0.042,0.725)	0.233(0.107,0.351)	0.28	0.08	77.78	22.22

r_G_ = genetic correlation between 2 phenotypes; r_E_ = unique environmental correlation between 2 phenotypes; c_G_ and c_E_ = genetic and unique environmental contribution to the correlation between 2 phenotypes, respectively; c_G = $r_{G}*\sqrt{A_{1}*A_{2}}$_; c_E = $r_{E}\;*\sqrt{E_{1}*E_{2}}$_; %_G_ and %_E_ = percentage of genetic and unique environmental contribution to the correlation between 2 phenotypes.

Abbreviations are the same as in Table 2.

Models were adjusted for age, sex, region, social economic status, smoking status, drinking status and physical activity.

## Discussion

Our results showed that significant associations and sex-specific effects existed between the measures of adiposity (BMII, WC, and PBF) and serum lipid, glucose, insulin and HOMA-IR levels. Analyses within MZ twin pairs allowed us to control for genetic influence thus the results were not confounded by genetic factors. Comparison of various adiposity measurements with levels of serum metabolites revealed that WC explained the largest fraction of variance in serum LDL-C, TG, TC and glucose concentrations than BMI while BMI performed best in explaining serum HDL-C, insulin and HOMA-IR levels. Based on genetic analyses, we found a strong genetic influence on adiposity measurements and serum metabolite levels separately and also on the adiposity-serum metabolite profile correlation. At the same time, environmental factors also contribute, particularly related to adiposity measurements and serum metabolite levels separately.

We confirmed the commonly accepted observation that BMI, WC, and PBF were associated with serum lipid, glucose, insulin and HOMA-IR levels in both sexes using a twin sample. On the contrary, LBM showed significant association on serum glucose, insulin and HOMA-IR levels but little on serum lipid levels which was consistent with previous studies [11, 23, 30]. This may be explained by different biological roles of FM and LBM. The LBM compartment is composed of organs and muscle, which are primarily responsible for whole-body glucose disposal and the contribution of LBM to the pathogenesis and development of insulin resistance has been declared [31]. The FM compartment is composed of adipose tissue, which functions in regulating whole-body energy metabolism, especially lipid storage and mobilization [32]. Taken together, our results were consistent with the close biological function of FM and LBM. When further controlling for genetic background using within MZ twin-pair analysis, the associations between adiposity measurements and serum lipid, glucose, insulin and HOMA-IR levels remained significant which suggested that these associations were independent of genetic influence. Analyses stratified by sex revealed that increasing BMI exerted a sex-specific deleterious role on insulin resistance, being it stronger within MZ male twins than within MZ female twins. This observation was consistent with a recent study which also reported a BMI-by-sex interaction on insulin resistance [33] and might help to explain the reason why women are more insulin sensitive and protected from diabetes at comparable BMI values than men. Besides, we also found the relationship between BMI and serum TG was stronger within MZ male twins than within MZ female twins. Therefore, these results were in line with the gender difference in the risk of CVD and T2MD. Using z-score transformed variables, the magnitudes of the β coefficients were comparable across different adiposity measures and the R^2^ values were also comparable in each regression model. WC had highest correlations and explained highest variance for most serum lipid levels. Therefore, our data tended to support that central obesity is considered a more important marker of lipid metabolic disturbances than total body adipose tissue. This observation was in accordance with previous studies which indicated that measures of central obesity were more sensitive for discriminating lipids abnormalities than BMI [34, 35]. Dyslipidemia in obesity may be explained by up-regulation of pro-inflammatory and pro-atherosclerotic mediators in dysfunctional adipose tissue, especially in subjects with high intra-abdominal deposition of visceral fat [36]. These data together reinforced the importance of assessing WC to identify individuals at a greater cardiometabolic risk. Besides, we found that BMI exerted greatest influence on serum insulin and HOMA-IR levels. Actually, progressive insulin resistance, may be the result of increased BMI, and not the other way around [37]. The mechanism can be elucidated by recent metabolomics studies which have found that BMI was positively associated with branched-chain amino acids (BCAA) which can interfere with insulin signaling and contribute to inducing insulin resistance [38].

Results from univariate genetic analyses showed that the overall heritability estimates for adiposity measurements and serum metabolite levels were moderate to high. These results indicated that these phenotypic variation was mostly due to genetic effects and this was consistent with the many univariate analyses published previously [24, 39–41]. Environment factors contributing to the variation of the phenotypes between individuals appeared to be mostly specific to individuals and not shared between family members. One possible reason may be that all our participants were adults and common environmental exposure shared by twin pairs was less marked than when the twin pairs were young [40].

As genetic factors account for a certain proportion of the population variance in adiposity measurements and serum metabolite levels, it is possible that genetic factors, at least in part, explain their associations. We found that a large proportion of phenotypic correlations between adiposity measurements and serum metabolite levels were explained by genetic factors. Previous twin studies which shown that phenotypic variation in most of the metabolic syndrome-related endophenotypes were genetic in origin [42, 43] supported our findings in bivariate genetic analyses. This indicated that these phenotypes, as expected, had genes in common. Besides, unique environmental factors also contributed to the associations between adiposity measurements and serum metabolite levels. The observation that both the genetic and unique environment factors influencing adiposity and serum metabolite levels were significantly correlated may support the possibility of causality, as expected under a causal model [44]. These data together suggested that the association between adiposity and serum metabolite levels were partly explained by the same genes, but also by the specific individual environmental factors. Since obesity-related non-genetic factors played a significant contribution to the dyslipidemia and pre-diabetic metabolite alterations, there is a potential for obesity related comorbidities prevention through modification of environmental factors. However, this study is not without its limitations. First, the cross-sectional design can only address associations and not casual relationships. Second, the possibility of residual confounding by unmeasured covariates cannot be excluded. Finally, a more direct and accurate assessment of adiposity measured by Dual-energy X-ray absorptiometry (DXA) or computed tomography (CT) may be less error-prone, however, many studies have observed the discriminatory capability of those simpler measures to be more robust than the measures derived from DXA or CT[45, 46].

## Conclusion

In summary, using a twin design to better control for confounders, we observed different associations between adiposity and serum metabolite profile and demonstrated that WC and BMI explained the largest fraction of variance in serum lipid profile and insulin resistance, respectively. Of these phenotypic correlations, 64–81% were attributed to genetic factors, whereas the remaining 19–36% were attributed to unique environmental factors. As genetic factors explained a large proportion of obesity-metabolite relation, further studies are needed to determine specific genes that influence both adiposity and serum metabolites. Furthermore, continued follow-up of this cohort would provide more insight into the relation and help to test the causality between adiposity and serum metabolite levels.

## Supporting Information

S1 Dataset Data of this manuscript. (XLSX)

S1 Table Pearson’s phenotypic correlations between different measures of body composition. (DOCX)

S2 Table Fixed-effect regression analyses of body composition measurements and serum metabolites within 235 MZ twin pairs stratified by gender (MZ male twins: 153 pairs; MZ female twins: 82 pairs). (DOCX)

S3 Table Best fitting models for all phenotypes in univariate genetic models. (DOCX)
