# Supplementary material for: Associations of Body Composition Measurements with Serum Lipid, Glucose and Insulin Profile: A Chinese Twin Study
Source: PLoS One. 2015 Nov 10;10(11):e0140595. doi: 10.1371/journal.pone.0140595 (PMC4640552; doi:10.1371/journal.pone.0140595)
Supplement: S1 Table — (DOCX) [file pone.0140595.s002.docx]

S1 Table. Pearson’s phenotypic correlations between different measures of body composition

|  | BMI | WC | PBF |
| --- | --- | --- | --- |
| BMI |  |  |  |
| WC | 0.825(0.763,0.867) |  |  |
| PBF | 0.602(0.566,0.641) | 0.466(0.394,0.499) |  |
| LBM | 0.504(0.445,0.556) | 0.544(0.478,0.605) | -0.266(-0.319,-0.211) |

BMI, body mass index; WC, waist circumference; PBF, percentage body fat; LBM, lean body mass.
