# Supplementary material for: Associations of Body Composition Measurements with Serum Lipid, Glucose and Insulin Profile: A Chinese Twin Study
Source: PLoS One. 2015 Nov 10;10(11):e0140595. doi: 10.1371/journal.pone.0140595 (PMC4640552; doi:10.1371/journal.pone.0140595)
Supplement: S2 Table — (DOCX) [file pone.0140595.s003.docx]

S2 Table. Fixed-effect regression analyses of body composition measurements and serum metabolites within 235 MZ twin pairs stratified by gender (MZ male twins:153 pairs; MZ female twins:82 pairs)

|  | HDL-C(mmol/L) |  | LDL-C(mmol/L) |  | TG(mmol/L) |  | TC(mmol/L) |  | Glucose (mmol/L) |  | Insulin(pmol/L) |  | HOMA-IR |
| --- | --- | --- | --- | --- | --- | --- | --- | --- | --- | --- | --- | --- | --- |
|  | *β*$(95\%CI)$ |  | *β*$(95\%CI)$ |  | *β*$(95\%CI)$ |  | *β*$(95\%CI)$ |  | *β*$(95\%CI)$ |  | *β*$(95\%CI)$ |  | *β*$(95\%CI)$ |
| Male |  | | | | | | | | | | | | |
| BMI(kg/m^2^) | -0.010(-0.015,-0.005)^***^ |  | 0.008(0.001,0.014) ^*^ |  | 0.038(0.026,0.051) ^***^ |  | 0.004(-.0000,0.008) |  | 0.005(0.002,0.008) ^*^ |  | 0.054(0.041,0.068) ^***^ |  | 0.059(0.045,0.074) ^***^ |
| WC(cm) | -0.002(-0.004,-.0003) ^**^ |  | 0.003(0.001,0.004) ^**^ |  | 0.011(0.006,0.016) ^***^ |  | 0.002(.0005,0.003) ^*^ |  | 0.002(.0006,0.002) ^*^ |  | 0.014(0.009,0.019) ^***^ |  | 0.016(0.010,0.021) ^***^ |
| PBF | -0.004(-0.006,-.0009) ^**^ |  | 0.004(0.009,0.007) ^*^ |  | 0.017(0.009,0.025) ^***^ |  | 0.002(.0005,0.004) ^*^ |  | 0.002(.0007,0.003) ^*^ |  | 0.023(0.015,0.031) ^***^ |  | 0.026(0.017,0.034) ^***^ |
| LBM(kg) | -0.006(-0.010,-0.003) ^***^ |  | 0.001(-0.003,0.005) |  | 0.017(0.008,0.025) ^**^ |  | .0001(-0.003,0.003) |  | 0.003(.0006,0.005) ^*^ |  | 0.027(0.017,0.036) ^***^ |  | 0.030(0.019,0.040) ^***^ |
| Female |  | | | | | | | | | | | | |
| BMI(kg/m^2^) | -0.005(-0.012,0.002) |  | 0.007(-.0002,0.015) |  | 0.014(-0.004,0.032) |  | 0.004(-0.002,0.010) |  | 0.004(-0.003,0.011) |  | 0.028(0.011,0.045) ^**^ |  | 0.032(0.016,0.049) ^***^ |
| WC(cm) | -.0006(-0.003,0.002) |  | 0.003(-.0004,0.006) |  | 0.006(.0000,0.012) ^*^ |  | 0.002(-.0003,0.004) |  | 0.002(-.0003,0.004) |  | 0.010(0.003,0.017) ^**^ |  | 0.012(0.005,0.018) ^***^ |
| PBF | .0001(-0.005,0.006) |  | 0.004(.0002,0.008) ^*^ |  | 0.005(-0.007,0.015) |  | 0.002(-.0004,0.005) |  | 0.001(-0.001,0.004) |  | 0.015(0.005,0.024) ^**^ |  | 0.016(0.006,0.026) ^***^ |
| LBM(kg) | -0.012(-0.021,-0.036) ^**^ |  | -0.001(-0.012,0.010) |  | 0.022(0.005,0.038) ^*^ |  | -0.001(-0.009,0.006) |  | .0000(-0.004,0.004) |  | 0.015(-0.006,0.036) |  | 0.015(-0.008,0.038) |

MZ, monozygotic; BMI, body mass index; WC, waist circumference; PBF, percentage body fat; LBM, lean body mass; HDL-C, high density lipoprotein cholesterol; LDL-C, low density lipoprotein cholesterol; TG, triglycerides; TC, total cholesterol; HOMA-IR, homeostasis model assessment of insulin resistance.

All regression models were adjusted for age, region, social economic status, smoking status, drinking status and physical activity.

*p<0.05

**p<0.01

***p<0.001
