# Supplementary material for: Associations of Body Composition Measurements with Serum Lipid, Glucose and Insulin Profile: A Chinese Twin Study
Source: PLoS One. 2015 Nov 10;10(11):e0140595. doi: 10.1371/journal.pone.0140595 (PMC4640552; doi:10.1371/journal.pone.0140595)
Supplement: S3 Table — (DOCX) [file pone.0140595.s004.docx]

S3 Table. Best fitting models for all phenotypes in univariate genetic models

| Phenotype | Model | A(95%CI) | E(95%CI) | -2LL | df | AIC | P |
| --- | --- | --- | --- | --- | --- | --- | --- |
| BMI | AE | 0.668(0.591,0.732) | 0.332(0.268,0.410) | 3391.841 | 655 | 2081.841 | 1.000 |
| WC | AE | 0.597(0.512,0.670) | 0.402(0.330,0.488) | 4803.420 | 655 | 3493.420 | 1.000 |
| PBF | AE | 0.669(0.594,0.730) | 0.331(0.270,0.406) | 4169.947 | 641 | 2887.947 | 1.000 |
| HDL-C | AE | 0.566(0.481,0.640) | 0.434(0.360,0.519) | -1363.433 | 655 | -2673.433 | 0.091 |
| LDL-C | AE | 0.663(0.589,0.724) | 0.337(0.276,0.411) | -1135.362 | 655 | -2455.362 | 1.000 |
| TG | AE | 0.520(0.426,0.602) | 0.480(0.398,0.574) | -75.904 | 655 | -1385.904 | 0.999 |
| TC | AE | 0.649(0.574,0.713) | 0.351(0.287,0.426) | -1577.913 | 655 | -2877.913 | 0.954 |
| Glucose | AE | 0.564(0.475,0.641) | 0.436(0.359,0.525) | -1731.554 | 655 | -3041.554 | 1.000 |
| Insulin | AE | 0.479(0.372,0.572) | 0.521(0.428,0.628) | -7.667 | 626 | -1259.667 | 1.000 |
| HOMA-IR | AE | 0.463(0.354,0.559) | 0.537(0.441,0.646) | 126.566 | 626 | -1125.434 | 1.000 |

A = Additive genetic; E = unique environment; –2LL =twice the negative log-likelihood; df = degree of freedom; P = χ2 test in model fitting; AIC, Akaike information criterion; BMI, body mass index; WC, waist circumference; PBF, percentage body fat; LBM, lean body mass; HDL-C, high density lipoprotein cholesterol; LDL-C, low density lipoprotein cholesterol; TG, triglycerides; TC, total cholesterol; HOMA-IR, homeostasis model assessment of insulin resistance.

Models were adjusted for age, sex, region, social economic status, smoking status, drinking status and physical activity.
